# Variations of Plasmid Content in *Rickettsia felis*

**DOI:** 10.1371/journal.pone.0002289

**Published:** 2008-05-28

**Authors:** Pierre-Edouard Fournier, Lokmane Belghazi, Catherine Robert, Khalid Elkarkouri, Allen L. Richards, Gilbert Greub, François Collyn, Motohiko Ogawa, Arantxa Portillo, Jose A. Oteo, Anna Psaroulaki, Idir Bitam, Didier Raoult

**Affiliations:** 1 Unité des rickettsies, IFR 48, CNRS-IRD UMR 6236, Faculté de médecine, Université de la Méditerranée, Marseille, France; 2 Naval Medical Research Center, Silver Spring, Maryland, United States of America; 3 Center for Research on Intracellular Bacteria, Institute of Microbiology, University Hospital Center and University of Lausanne, Lausanne, Switzerland; 4 Department of Virology I, National Institute of Infectious Diseases, Tokyo, Japan; 5 Hospital San Pedro- Centro de Investigación Biomédica de La Rioja (CIBIR), Área de Enfermedades Infecciosas, Logroño, Spain; 6 Laboratory of Clinical Bacteriology, Parasitology, Zoonoses and Geographical Medicine, University of Crete, Heraklion, Crete, Greece; 7 Unité d'Entomologie Médiale, Service d'Eco-Epidémiologie Parasitaire, Institut Pasteur, Alger, Algeria; Baylor College of Medicine, United States of America

## Abstract

**Background:**

Since its first detection, characterization of *R. felis* has been a matter of debate, mostly due to the contamination of an initial *R. felis* culture by *R. typhi*. However, the first stable culture of *R. felis* allowed its precise phenotypic and genotypic characterization, and demonstrated that this species belonged to the spotted fever group rickettsiae. Later, its genome sequence revealed the presence of two forms of the same plasmid, physically confirmed by biological data. In a recent article, Gillespie *et al.* (PLoS One. 2007;2(3):e266.) used a bioinformatic approach to refute the presence of the second plasmid form, and proposed the creation of a specific phylogenetic group for *R. felis*.

**Methodology/Principal Findings:**

In the present report, we, and five independent international laboratories confirmed unambiguously by PCR the presence of two plasmid forms in *R. felis* strain URRWXCal_2_^T^, but observed that the plasmid content of this species, from none to 2 plasmid forms, may depend on the culture passage history of the studied strain. We also demonstrated that *R. felis* does not cultivate in Vero cells at 37°C but generates plaques at 30°C. Finally, using a phylogenetic study based on 667 concatenated core genes, we demonstrated the position of *R. felis* within the spotted fever group.

**Significance:**

We demonstrated that *R. felis*, which unambiguously belongs to the spotted fever group rickettsiae, may contain up to two plasmid forms but this plasmid content is unstable.

## Introduction

*Rickettsia felis* (*R. felis*) was first detected in 1990 in American *Ctenocephalides felis* fleas using electron microscopy, and named the ELB agent after the Elward laboratory (Soquel, CA) where the flea colony was raised [1]. It was later detected by PCR in humans with a murine typhus-like illness [2]–[6]. Unfortunately, despite a first phylogenetic study clearly showing its classification within the spotted fever group (SFG) [7], confusion was brought by further reports, which attributed to the ELB agent, renamed *R. felis*, several characteristics of *R. typhi* [8]–[11]. It was later demonstrated that these data, including the protein profile [11], antigenic properties, growth conditions [8], [9], and antibiotic susceptibility [10], resulted from the contamination of a *R. felis* culture with *R. typhi* [12]. As a matter of fact, fleas can be infected by both *R. typhi* and *R. felis* [13], which may thus have been the source of contamination. In 2001, *R. felis* was cultivated from cat fleas at low temperature (28°C), and was established for the first time [2]. It was then deposited as strain URRWXCal_2_^T^ in two official collections: the American Type Culture Collection (ATCC VR-1525), and the Collection de Souches de l'Unité des Rickettsies (CSUR R121). Subsequently, another two teams were able to successfully grow *R. felis*, also at temperatures ≤32°C [14], [15] and not at 35–37°C as initially reported [9], [10], [12]. Phenotypic characterization of strain URRWXCal_2_^T^ demonstrated that its antibiotic susceptibility [16] and antigenic properties [17] classified it within the SFG. In 2005, we sequenced the genome of *R. felis* strain URRWXCal_2_^T^ [18] and demonstrated, using bioinformatics, pulsed field gel electrophoresis and southern blot, that this strain had two forms of the same plasmid, *i. e.*, a large, (pRF, 62-kb), and a small (pRFδ, 39-kb) forms. The pRF and PRFδ plasmids had identical sequences with the exception of an additional 24 ORFs in pRF. Then, we confirmed these results by PCR assays specifically targeting each plasmid [18]. The two plasmids were also detected in a collection of *Ctenocephalides felis* fleas from various locations. However, in a recent bioinformatic analysis of the *R. felis* genome sequence, Gillespie *et al.* questioned our results and proposed that the pRFδ plasmid was an artefact from genome assembly [19]. These authors based in part their conclusion on the results from Pornwiroon *et al.* who failed to detect the pRFδ plasmid in *R. felis* strain LSU [14], a strain cultivated in *C. felis* fleas at Louisiana State University [20]. In addition, on the basis of the analysis of a few genes, they proposed the classification of *R. felis* in a fourth phylogenetic lineage within the *Rickettsia* genus [19].

As our previous work had been carefully performed and experimentally confirmed, we believe that the conclusions of Gillespie *et al.* were not appropriate. Therefore, we asked five independent laboratories worldwide to check the presence of two plasmids in *R. felis*. We also evaluated the presence of both plasmids in various specimens and in cloned *R. felis*. Finally, we conducted a phylogenetic study based on 667 concatenated *Rickettsia* core protein-encoding genes, and tested new specimens.

## Results

### Presence of two plasmids in *R. felis* strain URRWXCal_2_^T^ and variation of plasmid content according to the passage history

**Figure 1:**
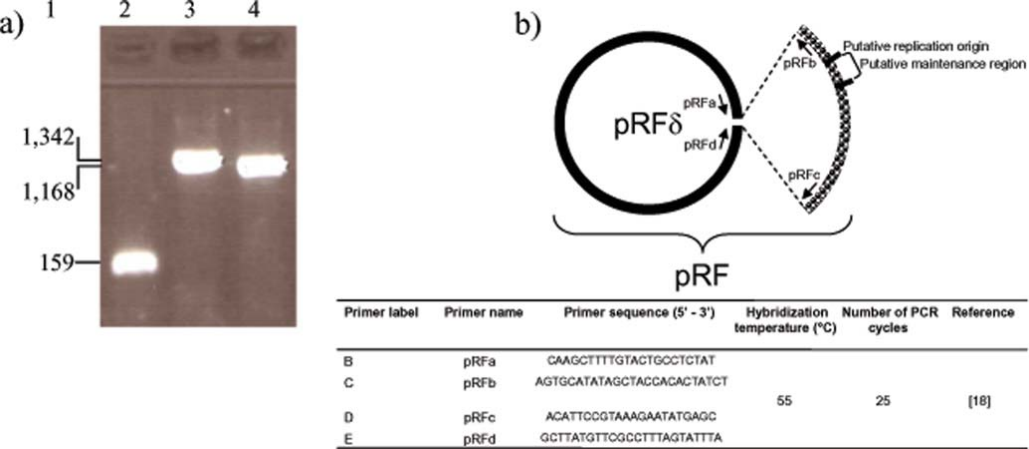
The *R. felis* plasmids. Specific PCR amplification of the two *R. felis* plasmid forms. Lane 1: Molecular size (bp); lane 2: pRFa-pRFb amplicon; lane 3: pRFc-pRFd amplicon; lane 4: pRFa-pRFd amplicon; b) Schematic representation of *R. felis* plasmids indicating the position of PCR primers used to amplify the pRF (pRFa/pRFb and pRFc/pRFd primer pairs) and pRFδ (pRFa/pRFd primer pair) plasmids.

**Figure 2:**
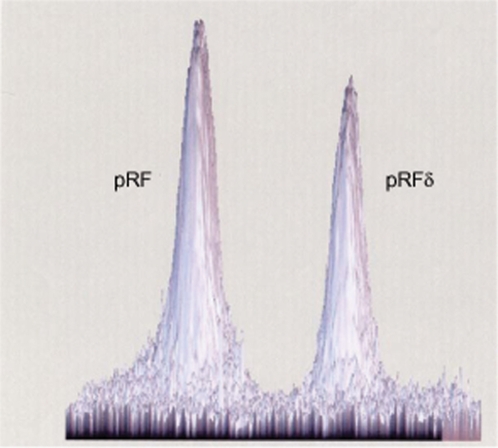
Cloning of *R. felis* cells using a plaque assay. White arrows show individual lysis plaques. Right, a lysis plaque was enlarged.

Using all four primer pairs, we obtained PCR products of the expected sizes from *R. felis* cultivated from the initial isolate [2]. Non-template controls were negative. The sequence obtained from the pRFa-pRFd amplicon was identical to GenBank accession number NC_007111, whereas pRFa-pRFb and pRFc-pRFd amplicons were identical to accession number NC_007110. Thus, we could reproduce our previously reported result and confirmed the presence of the two plasmids in *R. felis* strain URRWXCal_2_^T^. However, in order to obtain indisputable results, we proposed five independent laboratories worldwide to perform these PCR assays. To avoid any interpretation bias, we provided these laboratories with anonymized kits. All five laboratories obtained similar PCR results (Figure 1a, Table 1). A PCR product of the expected size was obtained for all 4 assays from DNA specimen 1, whereas for DNA specimens 2 and 3, only the AF-AR PCR provided a positive amplification. Negative controls were negative for all assays. Each of the five laboratories obtained a B-E PCR nucleotide sequence for *R. felis* identical to GenBank accession number NC_007111. Sequences were deposited in GenBank under accession numbers EU155007, EU056172, EU022170, EU040362, and EU017504 for laboratories L1 to L5, respectively. Further, when attempting to clone *R. felis* cells with a 40 cell culture passage history at various temperatures using the plaque assay, we obtained lysis plaques at 30°C but no culture at 37°C (Figure 2). Of 20 clones grown from single *R. felis* cells and individually collected, we obtained PCR products of the expected sizes for the two plasmid forms in 15 clones (75 %) but only the large plasmid form in the remaining five clones. In addition, only the small plasmid form was detected from the positive control DNA extracted from *R. felis* with a 50 cell culture passage history. These data demonstrated that the plasmid content of *R. felis* may vary according to culture history, with a small plasmid form being unstable. *R. felis* may exhibit from two (*R. felis* grown from initial frozen culture) to one plasmid form (*R. felis* with a 50 cell culture passages). Such a phenomenon was recently reported by Baldridge *et al.* who demonstrated that *R. peacockii* lost its plasmids during serial passage in cell culture [21].

**Table 1:** PCR results obtained by all five tester laboratories

DNA specimen (species)	PCR assay (primers)			
	B–E (pRFa-pRFd)	B-C (pRFa-pRFb)	D-E (pRFc-pRFd)	AF-AR
DNA1 (*R. felis*)	+	+	+	+
DNA2 (*R. conorii*)	-	-	-	+
DNA3 (*R. africae*)	-	-	-	+

**Figure 3:**
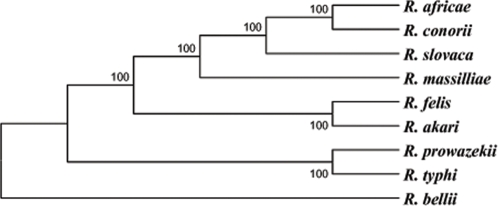
Determination of the *R. felis* plasmid ratio. The Southern blot obtained by hybridizing *R. felis* genomic DNA digested with PvuI and resolved by PFGE with probes specific for each plasmid form [18] was digitalized by transmission scanning (ImageScanner, Amersham Biosciences). The quantification of each labelled plasmid band was estimated by analysis with the ImageMaster 2D Platinium Version 6.0 software (Amersham Biosciences). The pRF and pRFδ spots represented 57% and 43%, respectively, of the hybridization intensity.

In their article, Gillespie *et al.* speculated, based on an *in silico* analysis, that the small plasmid form detected in *R. felis* was an artefact of genome assembly [19]. In our previous study, we were surprised to find two plasmid forms in *R. felis*, but found them both by pulsed field gel electrophoresis (Figure 3), southern blot, and genome assembly [18]. Moreover, we verified biologically that the small plasmid form can specifically be amplified by PCR, showing that the 2 plasmid forms exist both in cell culture and in the wild by amplification of *R. felis* DNA from *C. felis* fleas [18]. Therefore, the results that we and five independent laboratories obtained in the present study unambiguously confirm that our previous data did not result from a bioinformatic error and that two plasmid forms may co-exist in *R. felis* (Figure 1b). Moreover, by demonstrating in gels that the two plasmid forms were present in almost equal quantity in culture, we speculated that both forms may be present in a single *R. felis* cell (Figure 3). To explore this hypothesis, we cloned single *R. felis* cells and demonstrated that the small plasmid form lacked in 25 % of individual cells of this species. Baldridge *et al.* recently reported the detection of plasmids in five *Rickettsia* species, including two, *i.e.*, *R. peacockii* and *R. amblyommii* which had two plasmids [21]. These results, as well as ours, contradict the speculations of Gillespie *et al.* [19] that *R. felis* cannot have two plasmids.

### Variation of plasmid content in *R. felis* according to the strain

In addition to their bioinformatics analysis, Gillespie *et al.* based their conclusions on the results published by Pornwiroon *et al.* who did not detect the pRFδ plasmid form from *R. felis* strain LSU [14]. In the present study, the expert laboratory L1 detected the pRF but not the pRFδ plasmid form in *R. felis* strain LSU DNA provided by these authors, thus confirming their data [14]. Subsequently, we tested another *R. felis* strain, the RF2125 strain endemic in *Archaeopsylla erinacei* fleas from Algeria [22]. Although the pRF plasmid form was detected from 64 *A. erinacei*, we failed to detect the pRFδ plasmid form in any of these. Thus, in addition to culture conditions, the plasmid content of *R. felis* may vary from one strain to another. The former hypothesis is supported by the fact that the URRWXCal_2_^T^ strain may have one or two plasmid forms as demonstrated by our results, that the LSU strain, belonging to the same genotype, has one plasmid form [14], and that the RF2125 strain, genetically different from the other two strains (Table 2), may have one or no plasmid.

**Table 2:** Genetic variability of *R. felis* strain URRWXCal_2_^T^ compared to other studied strains

Strain	LSU^a^	LSU^b^	Algerian *A. erinacei* strain
URRWXCal_2_^T^	D^c^,^e^	I^d^,^e^	D^c^,^f^

a Sequences from the LSU strain were determined by Bouyer *et al.* [25];

b Sequences from the LSU strain were determined by Pornwiroon *et al.* [14];

c D = different genotype;

d I = Identical genotype;

e = *ompA* gene;

f = *gltA* gene.

### Phylogeny of *R. felis*

Using sequences from 15 chromosome-encoded proteins or from 21 conserved hypothetical proteins, Gillespie *et al.* proposed the creation of a fourth phylogenetic cluster within the *Rickettsia* genus, the “transitional group”, that contained *R. felis* [14]. However, the data produced by these authors do not provide any evidence that *R. felis* belongs to “a lineage distinct from other previously established taxonomic categories for rickettsiae”. As stated by the authors, their phylogenetic study is similar to other recently published rickettsial trees, which showed no evidence or need for the creation of a fourth lineage [23], [24]. This result even contradicts a previous article by the same team where *R. felis* was clearly associated with the SFG [25]. Initial speculations on an intermediate status of *R. felis*, with phenotypic properties similar to *R. typhi* but a genetic clustering with spotted fever group rickettsiae, resulted from studies that have not been reproduced [1], [8]–[11], [25], [26]. Moreover, the only strain recovered from this work was *R. typhi* and contamination was acknowledged in one of these four early papers [12]. By analysing ad hoc genes, one may also cluster *R. felis* and *R. bellii* because both species share unique characteristics among rickettsiae such as the largest chromosomes, or the presence of *tra* clusters and transposases [18], [23].

**Figure 4:**
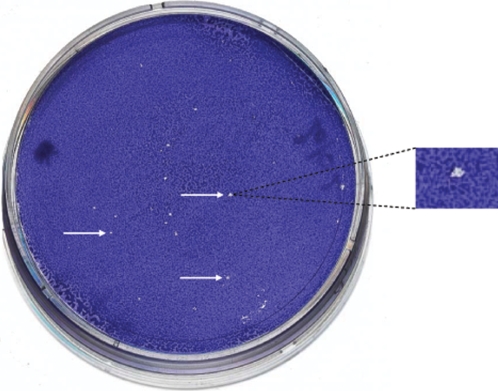
Phylogenetic tree inferred from the comparison of 667 concatenated *Rickettsia* core protein-coding genes using the maximum parsimony method. Bootstrap values are indicated at branch nodes. A similar topology was obtained using the Neighbor-Joining analysis method.

Moreover, the analysis of various genes that provided reliable phylogenetic organisations of rickettsiae demonstrated that *R. felis* was placed in a cluster also including *R. akari* and *R. australis* within the SFG [27]–[29]. As a matter of fact, *R. australis* is transmitted by ticks and has unambiguously been classified within the spotted fever group [30]. However, the reliability of phylogenetic studies based on selected genes may be impaired by a selection bias, in particular due to recombination or lateral gene transfer, as previously described for *Rickettsia* species [31]. In the most complete phylogenetic study of *Rickettsia* species performed to date, based on complete genome sequences, *R. felis* was clearly classified within the SFG [32]. Herein, with the same method as that used by Gillespie *et al.* [19], the phylogenetic tree that we inferred from the comparison of 667 concatenated core genes of *Rickettsia* unambiguously placed *R. felis* within the SFG (Figure 4). Therefore, our data do not demonstrate any necessity to create a new cluster. In addition, the distance between *R. felis* and other SFG species is not bigger than that between *R. helvetica*, another SFG species, and other SFG members for example [28].

### Correlation host vector-phylotype

The hypothesis that *Rickettsia* species have acquired virulence after the divergence of *R. bellii* and *R. canadensis* is misleading [19]. By omitting some data, one may build a simplified model of host-vector-rickettsia co-speciation, with *R. prowazekii* being associated with lice, *R. felis* with fleas and *R. rickettsii* with ticks, and subsequently determine which species is pathogenic or not. However, over the past 15 years, new data have contradicted this vision of rickettsiae [33]. As examples, *R. parkeri*, considered as non pathogenic for 65 years, was recently demonstrated to be a human pathogen [34]; *R. canadensis* is suspected epidemiologically and serologically to cause disease [35]; *R. bellii* has been shown to cause escharotic lesions when injected in guinea pigs [23] and may thus be pathogenic in humans. Finally, *Coxiella burnetii* (as *R. diasporica*), *R. africae* (as ESF agent), and *Legionella pneumophila* (as Tatlock agent) [36] were considered to be non-pathogenic rickettsiae in the past, before being recognized as human pathogens [33]. Recent findings demonstrated that the vector range of rickettsiae is not fully established [33]. For examples, *R. prowazekii* (a typical louse borne disease) was found in ticks in Africa and Mexico [37], [38] and was reported in lice and acarids from flying squirrels in the USA [39]. Similarly, *R. conorii* was reported to infect mites and lice [40], [41], and *R. bellii* was found in insects (unpublished data), although both species were believed to be strictly associated with ticks. Therefore, a simplification of the relationship between ecological niche, pathogenicity and phylotype is not possible, and these findings forced rickettsiologists to define more carefully rickettsiae as either pathogenic or of unknown pathogenicity. Moreover, by deliberately omitting SFG rickettsiae of unknown pathogenicity (such as the SFG species *R. montanensis*) the authors proposed in their Figure 1 a biased representation of what is currently known about rickettsial pathogenicity [19].

## Discussion

We were surprised that the only hypothesis produced by Gillespie *et al.* [19] to explain the discordance between our results, showing the presence of two plasmids in *R. felis* [18], and those of Pornwiroon *et al.* who could detect only one plasmid [14], was that the small plasmid form of *R. felis* was an artefact of our genome assembly. It is clear in other bacterial genera that the plasmid content may vary from one strain to another and that plasmids may not be stably maintained in culture [42], [43]. In our previous work, we had shown the presence of the two plasmid forms [18], and demonstrated in the present article that *R. felis* may have one, two or no plasmid, depending on the strain or the culture passage history. This plasmidic instability, which we also identified in *R. africae* (unpublished data), was also described recently in *R. peacockii* [21]. This phenomenon poses the problem of the significance of the genomic sequences of rickettsiae passaged many times in cell culture prior to sequencing, such as *R. prowazekii* or *R. conorii*.

Regarding the phylogenetic position of *R. felis*, the choice of genes for infering phylogenies should be extremely careful. For example, Woese clearly demonstrated discrepancies between the phylogenies obtained using the ribosomal operon and amino-acyl –tRNA synthetases [44]. This is mainly due to the fact that bacteria have a core set of conserved and inherited genes, which may be used to establish phylogenies reflecting their true evolution, and a set of genes acquired by lateral gene transfer or recombination, which may provide biased phylogenies [45]. By selecting a set of specific genes, Gillespie *et al.* [19] proposed the creation of a specific phylogenetic position for *R. felis*, between SFG and TG rickettsiae, contradicting biological data and their own work [25]. Moreover, *R. felis* may be subject to gene recombination with *R. typhi* as these two rickettsiae can meet in the same host flea. However, the current position of *R. felis* and its clustering with *R. australis* and *R. akari* is exactly the same as that defined in as early as 1999 [46]. Herein, we performed a unique phylogenetic study of *Rickettsia* species based on the concatenation of their core gene set and demonstrated unambiguously that *R. felis* belongs to the spotted fever group. Finally, classification of rickettsiae based on current knowledge of host specificity is not reliable.

We also demonstrated for the first time using a cloning method in Vero cells that *R. felis* does not grow at 37°C. This result confirms the fact that authors who initially reported a culture of this species at 37°C [8]–[11] did not grow the current strain of *R. felis* [47]. Their data may have resulted from a contamination with *R. typhi*, which they later acknowledged [12]. This also explains why phenotypic traits initially described for *R. felis* were similar to those of *R. typhi* [8]–[11].

We believe that it was important to clarify the status of *R. felis*. We clearly demonstrated that *R. felis* is a SFG rickettsia, that it does not grow at 37°C and that it has, without any possible doubt, two plasmids. Finally, we think that it would have been fair, since our *R. felis* strain is available, to check the presence of plasmids prior concluding that our data resulted from an error.

## Materials and Methods

### PCR detection of pRF and pRFδ plasmids

*R. felis* strain URRWXCal_2_^T^ kept frozen at -80°C since initial isolation was cultivated in XTC2 cells as previously described [47]. DNA was extracted from freshly cultivated *R. felis* using the QIAmp Tissue kit (Qiagen, Hilden, Germany). The pRFδ plasmid was detected using the primers pRFa and pRFd (expected size 1,168 bp, Figure 1). The pRF plasmid was detected using the primer pairs pRFa-pRFb (expected size 159 bp) and pRFc-pRFd (expected size 1,342 bp, Figure 1b). We used two negative controls, *i. e.*, sterile water and a PCR mix without DNA. The amplification conditions were as follows: 2.0 µL of DNA was mixed with 0.1 µL Platinum TaqDNA Pol High Fidelity polymerase (Invitrogen, Cergy, France), 2.5 µL High Fidelity PCR Buffer 10X, 0.5 µL of a 10mM dNTP mixture, 1 µL of 50 mM MgSO_4_, 0.5 µL of each primer (10mM), and 17.9 µL sterile water. Amplification conditions included an initial denaturation at 94°C for 2 min followed by 25 cycles comprised of 94°C for 30sec., 55°C for 30 sec., and 68°C for 2 min. PCR products were resolved in 1% agarose gels with ethidium bromide.

### Plasmid detection kit

We prepared a kit that contained DNA from *R. felis* strain URRWXCal_2_^T^ (DNA1), *R. conorii* strain Malish 7 (DNA2) and *R. africae* strain ESF-5 (DNA3) (Table 3) extracted using the QIAmp Tissue kit (Qiagen), and PCR primers. All reagents in this kit were anonymized. Primers pRFa, pRFb, pRFc, and pRFd were renamed B, C, D, and E, respectively (Figure 1b). The kit was sent to five independent expert laboratories worldwide, including laboratories located in the USA (laboratory L1), Switzerland (L2), Greece (L3), Spain (L4), and Japan (L5). In addition to the above-described DNA samples, each kit contained the primer pairs B-E specific for the pRFδ plasmid, B-C and D-E specific for the pRF plasmid (Figure 1b), and AF (5′-CCTATGGCTATTATGCTTGC-3′)–AR (5′-ATTGCAAAAAGTACAGTGAACA-3′) specific for the citrate synthase (*gltA*)-encoding gene. Each primer pair was tested on each of the three DNA specimens (Table 1). For each PCR assay, two negative controls were used, *i. e.*, sterile water and a PCR mix without DNA. For the B-E, B-C and D-E PCR assays, the amplification conditions were similar to those described above. For the AF-AR PCR assay, the amplification conditions were the following: 5.0 µL of DNA was mixed with 0.125 µL HotstarTaq Polymerase (Qiagen), 2.5 µL Buffer, 2.5 µL dNTP, 1 µL MgCl_2_, 0.5 µL of each primer, and 13.0 µL sterile water. Amplification conditions included an initial denaturation at 94°C for 15 min followed by 39 cycles comprised of 94°C for 1 min., 54°C for 30 sec., and 72°C for 2 min. The reaction was completed by a final elongation step at 72°C for 5 min.

**Table 3:** DNA specimens sent to expert laboratories

Anonymized DNA label	Species	Strain
DNA1	*R. felis*	URRWXCal_2_^T^ (ATCC VR1525)
DNA2	*R. conorii*	Malish 7^ T^ (ATCC VR613)
DNA3	*R. africae*	ESF-5^T^

### Variation of plasmid content in *R. felis*

K. Macaluso provided the expert laboratory L1 with DNA from five *R. felis*-positive (strain LSU) *C. felis* fleas [14]. This DNA was tested using the four above-described primer pairs and PCR conditions. We also tested 64 *Archaeopsylla erinacei* fleas from Algeria previously found to contain *R. felis* strain Rf2125 [22].

### Detection of pRF and pRFδ plasmids in single *R. felis* cells

Using tenfold dilutions of a suspension containing 10^4^ plaque forming units of *R. felis* strain URRWXCal_2_^T^ (40 cell culture passages since initial isolation), we inoculated Vero cells at 30°C and 37°C and performed a plaque assay as previously described [48]. Then, we collected individually 20 *R. felis* clones grown from single *R. felis* cells (Figure 2). DNA extraction from each clone, and plasmid detection, were performed as described above. As positive control, we used DNA extracted from our current *R. felis* strain URRWXCal_2_^T^ culture (50 cell culture passages).

### Phylogenetic analysis

To estimate the phylogenetic position of *R. felis* among *Rickettsia* species, we based our analysis on the 704 core protein-coding genes identified by Blanc *et al.* by comparison of 7 rickettsial genomes [32]. Of these genes, a total of 667 complete orthologous genes were found using the Blast software in the *R. slovaca* and *R. akari* genomes [49]. Subsequently, the amino acid sequences of these 667 proteins were concatenated for each genome and multiple alignment was performed using the Mafft software [50]. Gapped positions were removed. The maximum parsimony and neighbor joining trees were constructed using the MEGA 3.1 software [51]. Branching support was evaluated using the bootstrap method.
