# Correction: Variations of Plasmid Content in *Rickettsia felis*

**DOI:** 10.1371/annotation/6870e918-77d7-4ef5-ae5e-6cf994f9c169

**Published:** 2008-06-02

**Authors:** Pierre-Edouard Fournier, Lokmane Belghazi, Catherine Robert, Khalid Elkarkouri, Allen L. Richards, Gilbert Greub, François Collyn, Motohiko Ogawa, Arantxa Portillo, Jose A. Oteo, Anna Psaroulaki, Idir Bitam, Didier Raoult

Figures 2, 3, 4 and their legends are mismatched. Please view the correct figures with their correct legends here:

**Figure 2:**
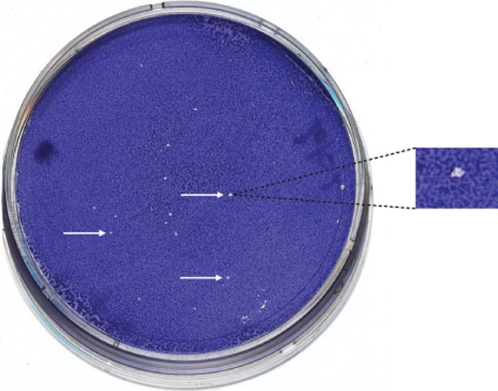
Cloning of *R. felis* cells using a plaque assay. White arrows show individual lysis plaques. Right, a lysis plaque was enlarged.

**Figure 3:**
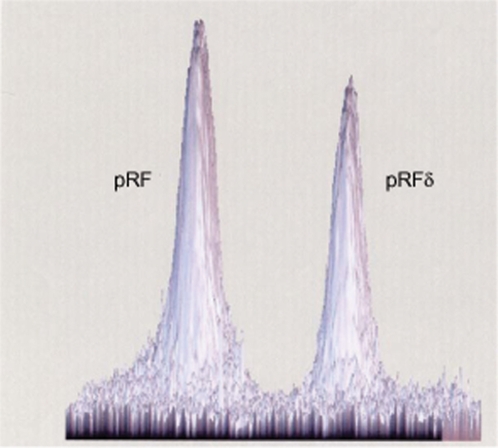
Determination of the *R. felis* plasmid ratio. The Southern blot obtained by hybridizing *R. felis* genomic DNA digested with PvuI and resolved by PFGE with probes specific for each plasmid form [18] was digitalized by transmission scanning (ImageScanner, Amersham Biosciences). The quantification of each labelled plasmid band was estimated by analysis with the ImageMaster 2D Platinium Version 6.0 software (Amersham Biosciences). The pRF and pRFδ spots represented 57% and 43%, respectively, of the hybridization intensity.

**Figure 4:**
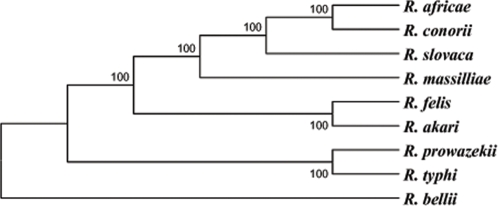
Phylogenetic tree inferred from the comparison of 667 concatenated *Rickettsia* core protein-coding genes using the maximum parsimony method. Bootstrap values are indicated at branch nodes. A similar topology was obtained using the Neighbor-Joining analysis method.

